# miR-26a-5p Attenuates Oxidative Stress and Inflammation in Diabetic Retinopathy through the USP14/NF-*κ*B Signaling Pathway

**DOI:** 10.1155/2024/1470898

**Published:** 2024-01-19

**Authors:** Jie Bian, Weizhong Ge, Zhengmei Jiang

**Affiliations:** Department of Ophthalmology, Yixing People's Hospital, The Affiliated Hospital of Jiangsu University, Yixing 214200, Jiangsu, China

## Abstract

**Purpose:**

Diabetic retinopathy (DR) is an ocular disease caused by diabetes and may lead to vision impairment and even blindness. Oxidative stress and inflammation are two key pathogenic factors of DR. Recently, regulatory roles of different microRNAs (miRNAs) in DR have been widely verified. miR-26a-5p has been confirmed to be a potential biomarker of DR. Nevertheless, the specific functions of miR-26a-5p in DR are still unclear.

**Methods:**

Primary cultured mouse retinal Müller cells in exposure to high glucose (HG) were used to establish an *in vitro* DR model. Müller cells were identified via morphology observation under phase contrast microscope and fluorescence staining for glutamine synthetase. The *in vivo* animal models for DR were constructed using streptozotocin-induced diabetic C57BL/6 mice. Western blotting was performed to quantify cytochrome c protein level in the cytoplasm and mitochondria of Müller cells and to measure protein levels of glial fibrillary acidic protein (GFAP), ubiquitin-specific peptidase 14 (USP14), as well as factors associated with NF-*κ*B signaling (p-I*κ*B*α*, I*κ*B*α*, p-p65, and p65) in Müller cells or murine retinal tissues. ROS production was detected by CM-H2DCFDA staining, and the concentration of oxidative stress markers (MDA, SOD, and CAT) was estimated by using corresponding commercial kits. Quantification of mRNA expression was conducted by RT-qPCR analysis. The concentration of proinflammatory factors (TNF-*α*, IL-1*β,* and IL-6) was evaluated by ELISA. Hematoxylin-eosin staining for murine retinal tissues was performed for histopathological analysis. Immunofluorescence staining was conducted to determine NF-*κ*B p65 nuclear translocation in Müller cells. Furthermore, the interaction between miR-26a-5p and USP14 was verified via the luciferase reporter assays.

**Results:**

HG stimulation contributed to Müller cell dysfunction by inducing inflammation, oxidative injury, and mitochondrial damage to Müller cells. miR-26a-5p was downregulated in Müller cells under HG condition, and overexpression of miR-26a-5p relieved HG-induced Müller cell dysfunction. Moreover, miR-26a-5p targeted USP14 and inversely regulated USP14 expression. Additionally, HG-evoked activation of NF-*κ*B signaling was suppressed by USP14 knockdown or miR-26a-5p upregulation. Rescue assays showed that the protective impact of miR-26a-5p upregulation against HG-induced Müller cell dysfunction was reversed by USP14 overexpression. Furthermore, USP14 upregulation and activation of NF-*κ*B signaling in the retinas of DR mice were detected in animal experiments. Injection with miR-26a-5p agomir improved retinal histopathological injury and weakened the concentration of proinflammatory cytokines and oxidative stress markers in the retinas of DR mice.

**Conclusion:**

miR-26a-5p inhibits oxidative stress and inflammation in DR progression by targeting USP14 and inactivating the NF-*κ*B signaling pathway.

## 1. Introduction

Diabetic retinopathy (DR) is a chronic diabetic complication characterized by an ocular disorder that can lead to impaired vision and even blindness. DR is seriously threatening the eye health of people worldwide [1] and partly results from unhealthy eating habits and lifestyles nowadays [2]. Clinically, fundus lesions in DR are manifested as retinal detachment, macular edema, vitreous proliferation, neovascularization, and cotton patch. At present, the first-line therapy for diabetic macular edema, a common cause of vision loss in patients with DR, is anti-VEGF administration. However, some patients with DR show tachyphylaxis or refractoriness to the repeated injection of anti-VEGF drugs [3]. Therefore, finding preventive and therapeutic methods to control DR is of great urgency.

Oxidative stress and inflammatory response induced by chronic hyperglycemia are key factors for the occurrence and progression of DR [4]. Oxidative stress is the consequence of the imbalance between the release of endogenous antioxidant factors and the release of reactive oxygen species (ROS) [5]. The excessive release of ROS can result in the damage of tissues around and in retinal vessels, thereby leading to DR [6]. Moreover, hyperglycemia-induced oxidative stress can dramatically enhance inflammation and angiogenesis [6]. Emerging evidence has manifested the crucial role of ROS in the activation of a typical proinflammatory signaling, i.e., nuclear factor-kappa B (NF-*κ*B) signaling [7]. Once NF-*κ*B is activated, nuclear translocation of NF-*κ*B occurs and the transcription of proinflammatory factors happens. Interleukin-1*β* (IL-1*β*), interleukin 6 (IL-6), and tumor necrosis factor-*α* (TNF-*α*) are common proinflammatory factors [8]. As the main glial cells in the retinas, Müller cells undergo oxidative injury and show reactive phenotypes under hyperglycemic conditions [9]. Activation of Müller cells is manifested as the upregulation of glial fibrillary acidic protein (GFAP) expression and the subsequent release of proinflammatory cytokines [10]. Thus, inhibition of the GFAP, oxidative stress, and inflammatory response in Müller cells under hyperglycemic conditions may prevent the pathological process of DR [11].

MicroRNAs (miRNAs) are small, endogenous, noncoding RNAs of ∼22 to 26 nucleotides in length. The posttranscriptional regulation is a key function of miRNAs [12]. miRNAs interact with the 3′ untranslated regions (UTRs) of target genes, thereby repressing the translation or promoting the degradation of mRNAs [13]. Many studies have demonstrated that miRNAs can modulate various biological processes, including cell proliferation, metabolism, and organ development [14, 15]. miRNAs have been considered as promising candidates for targeted therapeutic approaches to DR [16, 17]. Emerging evidence has identified the close association of miRNAs with the occurrence or development of DR. For example, depleted miR-29a/b leads to Müller cell dysfunction to exacerbate DR by targeting mRNA forkhead box protein O4 [18]. Overexpressed miR-18a-3p alleviates blood-retinal barrier disruption in DR mice and mitigates inflammatory injury in Müller cells under high glucose conditions by targeting the gene bone morphogenetic protein 4 [19]. miR-138-5p displays low expression in DR and its upregulation represses the proliferative potential of retinal capillary endothelial cells by interacting with mRNA neuro-oncological ventral antigen 1 [20]. miR-26a-5p is a well-known miRNA and its regulatory role has been demonstrated in different human diseases, including diabetes and diabetic complications. For example, miR-26a-5p attenuates myocardial injury in diabetic rats by downregulating phosphatase and tensin homolog [21]. Long noncoding RNA and small nucleolar RNA host gene 5 target miR-26a-5p to promote renal tubular cell damage by accelerating cell apoptosis, oxidative stress, and inflammation in diabetic nephropathy [22]. Upregulation of miR-26a-5p prevents inflammatory response in tubular epithelial cells by binding with target gene CHAC1 and inhibiting NF-*κ*B signaling, thereby attenuating diabetic kidney disease [23]. Importantly, a previous study has identified that miR-26a-5p suppresses retinal neuronal cell death to prevent retinal impairment by interacting with mRNA phosphatase and tensin homolog in diabetic mice [24]. Another study has pointed out that circulating miR-26a-5p is downregulated in nonproliferative DR, suggesting its potential involvement in retinal neurodegeneration at an early stage [25]. Nevertheless, the molecular mechanism mediated by miR-26a-5p in DR and its functions in Müller cells need further investigation.

Ubiquitin-specific peptidase 14 (USP14), a ubiquitin-specific protease associated with the proteasome, exerts essential functions in inflammation, cellular functions, neurodegenerative diseases, and tumor development [26]. It is reported that USP14 is highly expressed in DR patients and can alter the functions of Müller cells treated with high glucose by enhancing I*κ*Bɑ deubiquitination and degradation, thereby promoting I*κ*Bɑ phosphorylation and NF-*κ*B activation [27]. However, the miR-26a-5p/USP14 regulatory network in DR has not been explored.

In the current work, the *in vitro* and *in vivo* models for DR are established using high glucose- (HG-) stimulated Müller cells and streptozotocin-induced diabetic mice to investigate the biological role and molecular mechanism of miR-26a-5p in DR. Our study might provide promising insight into potential therapeutical strategies for DR treatment.

## 2. Materials and Methods

### 2.1. Cell Culture

Mouse primary retinal Müller cells were isolated from 3-day-old newborn C57BL/6 mice (Vital River, Beijing, China), and Müller cells were identified and extracted according to the previous description [28]. Retinal Müller cells were maintained in DMEM containing 10% fetal bovine serum (Gibco, USA), 100 U/ml penicillin, and 100 mg/ml streptomycin (Gibco) with 5% CO_2_ at 37°C.

### 2.2. Cell Identification

Müller cells were identified through morphology observation under a phase contrast microscope. Additionally, fluorescent staining for glutamine synthetase (GS), a specific enzyme of Müller cells in the retina, was also performed for cell identification. A fluorescence microscope (Leica, Germany) was utilized for visualization of the fluorescence of Müller cells.

### 2.3. Cell Treatment

When cell confluence reached 80%, Müller cells were subjected to high glucose (HG) or normal glucose (NG) treatment. To mimic the diabetic environment, 33.3 mM of glucose (HG) was utilized to stimulate Müller cells for 24 h. Müller cells treated with 5 mM of glucose (normal glucose) for 24 h served as the control group.

### 2.4. Cell Transfection

The miR-26a-5p mimics were required to overexpress miR-26a-5p, and the control group was labeled as negative control (NC) mimics (sequence: UUCUCCGAACGUGUCACGUTT). The coding region of USP14 was inserted into pcDNA3.1 vectors to overexpress USP14, with empty vectors as the control. When the cell confluence reached 80%, 50 nM of pcDNA3.1/USP14, 50 nM of miR-26a-5p mimics, or 50 nM of corresponding negative controls were transfected into Müller cells using Lipofectamine 2000 (Invitrogen, CA, USA) at 37°C for 48 h following the supplier's guidance. The transfection efficiency was examined by RT-qPCR after 48 h. All plasmids were synthesized by GenePharma (Shanghai, China). Sequences for NC mimics/miR-26a-5p mimics and sh-NC/sh-USP14 are provided in Table 1.

### 2.5. Immunofluorescence Staining

In brief, cultured Müller cells were fixed with 4% buffered paraformaldehyde and then washed with PBS for 5 min. Then, the cells were incubated overnight at 4°C with primary antibodies against NF-*κ*B phospho-p65 (ab222494, Abcam), followed by incubation with a FITC-labeled anti-goat secondary antibody. At room temperature, the cell nuclei were counterstained with DAPI (Beyotime, Shanghai, China) for 20 min. Images were examined under a fluorescence microscope (Olympus, Japan).

### 2.6. Establishment of Animal Models

A total of 40 male C57BL/6 mice (8 weeks old) were purchased from Vital River (Beijing, China). All mice were kept in standard pathogen-free conditions (12 h/12 h light/dark cycle, 24.0 ± 0.5°C) with free access to water and food. The experiments were performed in compliance with the Animal Ethics Committee of Yixing Hospital affiliated to Jiangsu University. The mice were then divided into four groups (*n* = 10 in each group): normal, DR, DR + NC-agomir, and DR + miR-26a-5p agomir. The diabetic mice models were established as previously described [29]. Specifically, mice were fasted for 12 hours before streptozotocin (STZ) injection. Then, the mice in the three DR groups (DR, DR + NC-agomir, and DR + miR-26a-5p agomir) received 50 mg of STZ (CAS no. 18883-66-4, HPLC purity ≥98%; Wuhan Tianzhi Biotechnology Co., LTD, Wuhan, China) once per day for 5 consecutive days by intraperitoneal injection. Mice with a blood glucose level of over 16.7 mmol/l were regarded as diabetic and were subjected to the following experiments [29]. Mice in the normal group were intraperitoneally injected with the same amount of citrate buffer. One week after successful modeling, mice in the DR + miR-26a-5p group were intravenously injected with 10 nmol of miR-26a-5p agomir (RiboBio) in 200 *μ*l of saline via the tail vein and mice in the DR + NC-agomir group received an equivalent amount of NC-agomir in the same way. Twenty-four hours after the operation, the mice were subsequently euthanized, and the retinal tissue samples were harvested for further analysis. All experiments were conducted in accordance with the National Institutes of Health Guide for the Care and Use of Laboratory Animals.

### 2.7. Hematoxylin and Eosin (H&E) Staining

After the mice were sacrificed, the retinas of different groups were resected and fixed with 4% paraformaldehyde. The fixed tissues were embedded in paraffin and sectioned (4 *μ*m thick) for dewaxing and dehydration. Tissue sections were then stained with hematoxylin and eosin following standard procedures, dehydrated with ethanol, cleared with xylene, and mounted in neutral balsam. The morphological changes of the samples were observed by using an inverted microscope (Olympus, Japan).

### 2.8. Measurement of ROS Levels

The levels of ROS in Müller cells were measured by CM-H_2_DCFDA (Thermo Fisher Scientific). Müller cells with the abovementioned treatments were seeded on 24-well plates. Then, the cells were incubated with 10 *μ*M of CM-H_2_DCFDA for half an hour at 37°C. Images were obtained by utilizing a confocal laser fluorescence microscope (Molecular Devices, Sunnyvale, CA, USA), and the fluorescence intensity was measured by using ImageJ software.

### 2.9. RT-qPCR

Total RNAs isolated from Müller cells by TRIzol reagent (Invitrogen) were reverse transcribed to complementary DNA using a reverse transcription cDNA synthesis kit (Vazyme, China), and then the SYBR Premix Ex Taq^TM^ Kit (Takara Biomedical Technology, Beijing, China) was utilized for RT-qPCR analysis on a 7900HT Fast Real-Time PCR System (Applied Biosystems). Sequences for the primers used are presented in Table 2. miR-26a-5p expression was normalized to U6. USP14 level was standardized to GAPDH. The relative RNA expression is calculated with the 2^−∆∆*Ct*^ method [30].

### 2.10. Luciferase Reporter Assay

The binding site between miR-143-3p and USP14 3′UTR was predicted with the starBase website (https://starbase.sysu.edu.cn/). The USP14 3′UTR fragment containing the binding sequence to miR-26a-5p was inserted into the pmirGLO vector (Promega) to form a wild-type luciferase reporter (USP14-Wt). USP14 3′UTR-Mut was formed using the mutated seed site. USP14 3′UTR Wt/Mut was transfected into HEK293T cells with miR-26a-5p mimics/NC mimics using Lipofectamine 2000 (Invitrogen). After 48 h of transfection, the luciferase activity (Firefly/Renilla) of each group was assessed using the Luciferase Reporter Assay System (Promega) [31].

### 2.11. Western Blotting

Total protein was extracted from cultured Müller cells and mouse retinas with RIPA lysis buffer (Thermo Fisher Scientific, Waltham, MA, USA). Cytoplasmic and nuclear proteins were extracted from cells using the PARIS Kit (#AM1556, Life Technologies, USA) based on the manufacturer's suggestions. Cytoplasmic and mitochondrial proteins were extracted from cells using a Cell Mitochondria Isolation Kit (#C3601, Beyotime, Shanghai, China) based on the manufacturer's suggestions. The protein concentration was measured by a BCA assay kit (#P0012S, Beyotime, China). Equal amounts of protein samples (20 *μ*g) were separated by 10% SDS-PAGE and blotted on polyvinylidene fluoride membranes (Millipore, Billerica, MA, USA). After blocking with 5% defatted milk and rinsing with tris-buffered saline containing 0.1% Tween 20 (TBST) buffer, the membranes were incubated at 4°C overnight with primary antibodies against USP14 (ab137433, 1 : 1000, Abcam, Cambridge, MA, USA), I*κ*B*α* (1 : 1000, #4812, Cell Signaling Technology, CST, Boston, MA, USA), phospho (p)-I*κ*B*α* (#2859, 1 : 1000, CST), cytochrome c (1 : 1000, #4272, CST), COXIV (1 : 1000, #4844, CST), GFAP (1 : 1000, #80788, CST), Histone H3 (1 : 2000, #4499, CST), NF-*κ*B *p*65 (1 : 1000, #8242, CST), NF-*κ*B phospho-p65 (1 : 1000, #3033, CST), IL-6 (1: 1000, #12912, CST), IL-1*β* (1: 1000, #12703, CST), TNF-*α* (1: 1000, #11948, CST), and GAPDH (1: 1000, #5174, CST). Afterwards, the membranes were incubated with the secondary antibody (ab7090, Abcam) at room temperature for 2 h. Eventually, protein bands were visualized with an enhanced chemiluminescence kit (Fdbio Science,Hangzhou, China) and the intensity was quantified with ImageJ software (GE Healthcare, Beijing, China). GAPDH, COXIV, and Histone H3 were used as cytoplasmic, mitochondrial, and nuclear internal controls, respectively.

### 2.12. Detection of Oxidative Stress Markers

The homogenized retinal tissues or Müller cells were subjected to centrifugation to obtain the supernatants. The levels of SOD (superoxide dismutase), CAT (catalase), and MDA (malondialdehyde) in the supernatants were estimated by a SOD assay kit (#S0101M, Beyotime, China), a CAT assay kit (#CAT100, Sigma-Aldrich, USA), and a MDA assay kit (#S0131M, Beyotime) according to the suppliers' instructions, respectively.

### 2.13. Enzyme-Linked Immunosorbent Assay (ELISA)

Measurement of the IL-1*β*, IL-6, VEGF, and TNF-*α* in the supernatants of homogenized retinal tissues or Müller cells was performed in the collected supernatants by utilizing corresponding kits based on the suppliers' suggestions, respectively.

### 2.14. Statistical Analysis

SPSS 18.0 software (SPSS Inc., USA) was used for data analyses. Experimental data are shown as the mean ± standard error of the mean (SEM). Differences between the two groups were tested by using an independent sample *t*-test, and multigroup comparisons were evaluated by using a one-way analysis of variance, followed by Tukey's post hoc test. A *P* value of less than 0.05 was set as the threshold for statistical significance.

## 3. Results

### 3.1. Identification of Mouse Primary Retinal Müller Cells

**Figure 1:**
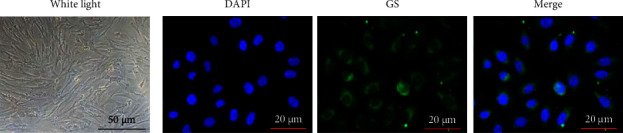
Identification of mouse primary retinal Müller cells. (a) Müller cell morphology under a phase contrast microscope. (b) Fluorescence staining for glutamine synthetase (GS) was performed for cell identification.

A phase contrast microscope showed that Müller cells were oval-shaped and in a pale color with a big nucleus and abundant cytoplasm (Figure 1(a)). Additionally, there are long pyramidal projections at two ends of the cell body (Figure 1(a)). The results of GS staining revealed that nearly all the cells were stained with GS which is a Müller cell-specific enzyme in the retina (Figure 1(b)). The findings confirmed that the cells used in the study are Müller cells.

### 3.2. miR-26a-5p Overexpression Relieves HG-Induced Müller Cell Dysfunction

**Figure 2:**
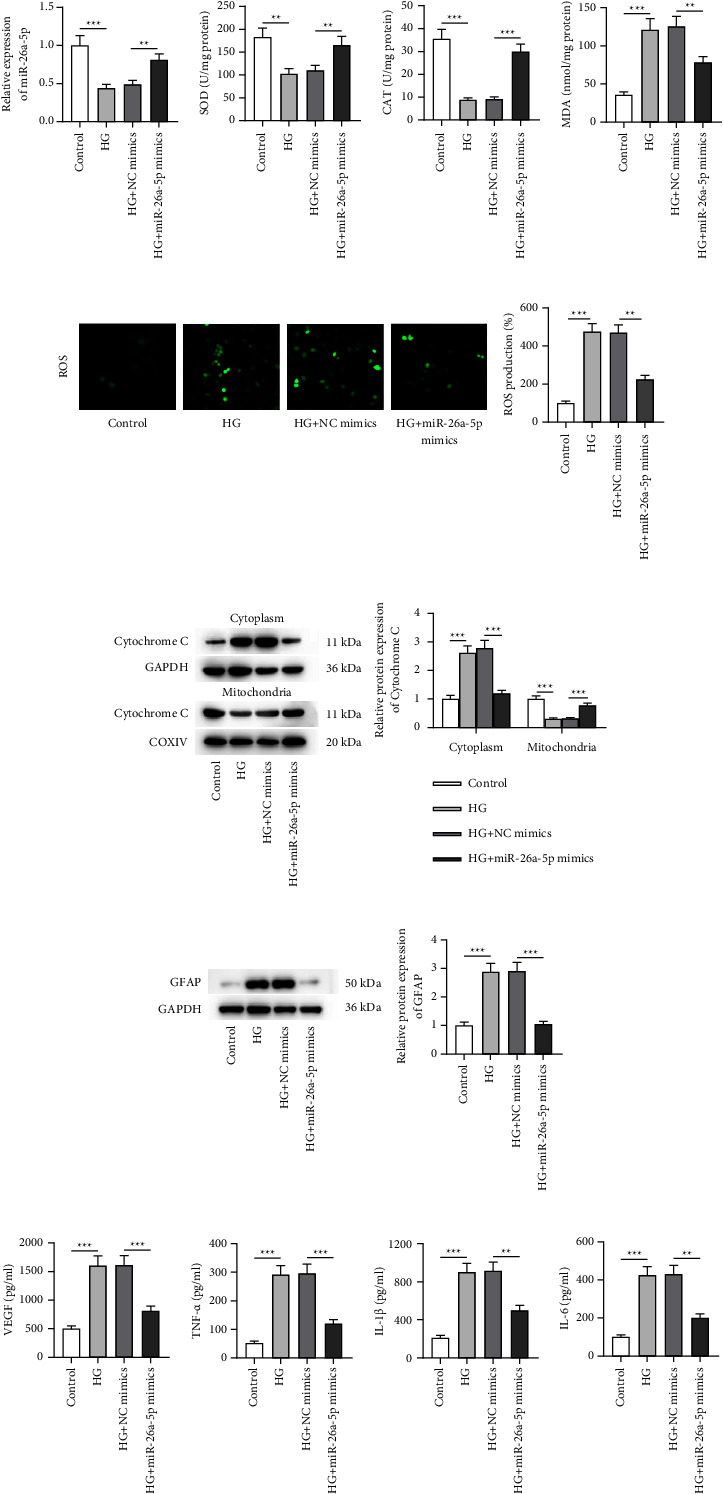
miR-26a-5p overexpression relieves HG-induced Müller cell dysfunction. (a) RT-qPCR was performed to measure miR-26a-5p expression in retinal cells with or without HG treatment and to evaluate the transfection efficiency of miR-26a-5p mimics in HG-exposed Müller cells. (b–d) SOD, CAT, and MDA levels in Müller cells of four groups (control, HG, HG + NC mimics, and HG + miR-26a-5p mimics groups) were measured using specific commercial kits. (e) CM-H_2_DCFDA staining was conducted to evaluate ROS levels in Müller cells of the control, HG, HG + NC mimics, and HG + miR-26a-5p mimics groups. (f) Western blotting was performed to quantify cytochrome c protein levels in the cytoplasm and mitochondria of Müller cells in the control, HG, HG + NC mimics, and HG + miR-26a-5p mimics groups. (g) Western blotting was performed to quantify GFAP protein expression in Müller cells of the control, HG, HG + NC mimics, and HG + miR-26a-5p mimics groups. (h–k) ELISA was conducted to measure (h) VEGF level and (i–k) the concentration of proinflammatory cytokines (TNF-*α*, IL-1*β,* and IL-6) in Müller cells of the control, HG, HG + NC mimics, and HG + miR-26a-5p mimics groups. ^*∗∗*^*p* < 0.01 and ^*∗∗∗*^*p* < 0.001.

An *in vitro* cell model for DR was established by stimulating Müller cells with a high concentration of glucose. Results from RT-qPCR illustrated that miR-26a-5p was downregulated in HG-treated Müller cells compared to that in Müller cells without any treatment (Figure 2(a)), implying that miR-26a-5p might substantially participate in DR. To examine the functions of miR-26a-5p in HG-induced Müller cell injury models, miR-26a-5p was overexpressed in HG-treated Müller cells via transfection of miR-26a-5p mimics, as demonstrated by RT-qPCR (Figure 2(a)). The outcome of Figures 2(b–d) denoted that the activities of antioxidant enzymes (SOD and CAT) were decreased and the concentration of prooxidant MDA was increased after HG treatment, while miR-26a-5p overexpression reversed these changes. CM-H_2_DCFDA staining assay recorded that miR-26a-5p mimics inhibit HG-stimulated ROS production (Figure 2(e)). The abovementioned results indicated that miR-26a-5p upregulation mitigates HG-evoked oxidative stress in Müller cells. Moreover, cytochrome c release from the mitochondria into the cytosol is a key induction of cell apoptosis [32]. The outcome of western blotting demonstrated HG-induced cytochrome c release from the mitochondria to cytosol, which was counteracted by miR-26a-5p enhancement (Figure 2(f)), implying that upregulated miR-26a-5p suppresses HG-triggered Müller cell apoptosis. Furthermore, retinal gliosis characterized by upregulation of GFAP and VEGF in Müller cells is an important pathological characteristic of DR [33]. As western blotting and ELISA denoted, miR-26a-5p augmentation offset the promoting effect of HG on GFAP protein expression and VEGF release in Müller cells (Figures 2(g and h)), which implied that HG-induced Müller cell gliosis was mitigated by upregulating miR-26a-5p. Additionally, HG stimulation caused Müller cell inflammation as evidenced by the increase in the concentration of proinflammatory cytokines (TNF‐*α*, IL‐1*β,* and IL‐6) in the HG group, but miR-26a-5p overexpression reversed the alterations in the release of inflammatory cytokines (Figures 2(i–k)). Overall, miR-26a-5p overexpression alleviated HG-induced Müller cell dysfunction.

### 3.3. HG-Evoked Activation of NF-*κ*B Signaling Was Suppressed by USP14 Knockdown or miR-26a-5p Elevation

**Figure 3:**
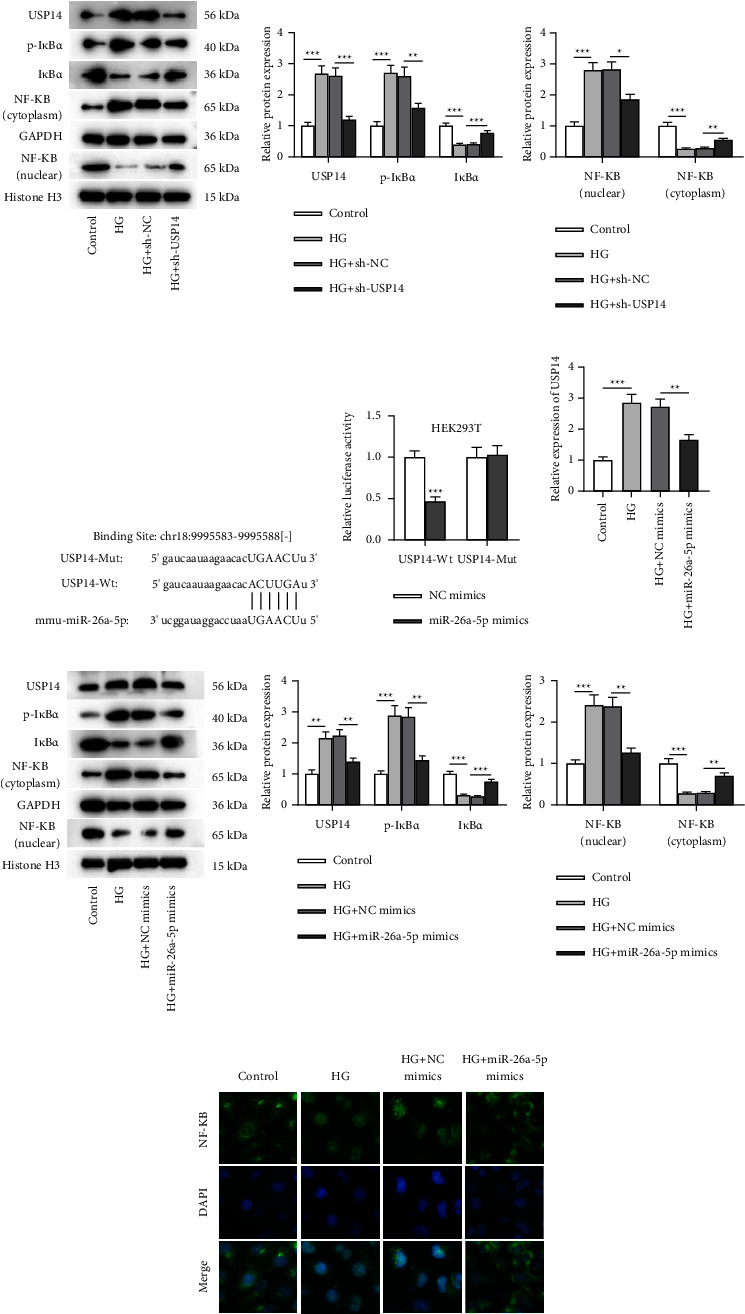
HG-evoked activation of NF-*κ*B signaling was suppressed by USP14 knockdown or miR-26a-5p elevation. (a) Western blotting was conducted to quantify protein levels of USP14, p-I*κ*B*α*, I*κ*B*α*, NF-*κ*B (cytoplasm), GAPDH, NF-*κ*B (nuclear), and histone H3 in Müller cells of the control, HG, HG + sh-NC, and HG + sh-USP14 groups. (b) The binding site between miR-26a-5p and USP14 3′UTR (from starBase). (c) Luciferase reporter assay was carried out for confirming the interaction between miR-26a-5p and USP14 3′UTR. (d) RT-qPCR was performed to detect USP14 expression in Müller cells of the control, HG, HG + NC mimics, and HG + miR-26a-5p mimics groups. (e) Western blotting was conducted for quantifying the protein levels of USP14, p-I*κ*B*α*, I*κ*B*α*, NF-*κ*B (cytoplasm), GAPDH, NF-*κ*B (nuclear), and histone H3 in Müller cells of the control, HG, HG + NC mimics, and HG + miR-26a-5p mimics groups. (f) Immunofluorescence staining was performed to visualize the intensity of NF-*κ*B in Müller cells of the control, HG, HG + NC mimics, and HG + miR-26a-5p mimics groups. ^*∗*^*p* < 0.05, ^*∗∗*^*p* < 0.01, and ^*∗∗∗*^*p* < 0.001.

Many reports have mentioned that USP14 can activate NF-*κ*B signaling. Additionally, it is well documented that NF-*κ*B activation promotes the progression of DR. Hence, USP14 protein expression in the *in vitro* cell model was quantified. It is shown that the USP14 protein level was increased after HG stimulation (Figure 3(a)), suggesting the potential involvement of USP14 in DR. Subsequently, the relationship of miR-26a-5p, USP14, and NF-*κ*B signaling in HG-stimulated Müller cells was figured out. USP14 was downregulated via transfecting sh-USP14 into Müller cells under HG condition (Figure 3(a)). Quantification of the protein levels of p-I*κ*B*α*, I*κ*B*α*, NF-*κ*B (nuclear), and NF-*κ*B (cytoplasm) was also conducted via western blotting. HG exposure triggered the increase in p-I*κ*B*α* and NF-*κ*B (nuclear) levels and the decrease in I*κ*B*α* and NF-*κ*B (cytoplasm) protein levels in Müller cells. However, these changes were reversed by the interference of USP14. The abovementioned findings confirm that silenced USP14 represses HG-mediated activation of NF-*κ*B signaling in Müller cells. A binding site between miR-26a-5p and USP14 3′UTR is provided by a bioinformatics tool, starBase (Figure 3(b)). To verify the interaction of the two RNAs, the luciferase reporter plasmid USP14-Wt/Mut was constructed and transfected into HEK293T cells together with miR-26a-5p mimics/NC mimics. Data showed that miR-26a-5p mimics caused a reduction of luciferase activity in the USP14-Wt group, but miR-26a-5p mimics had little impact on the activity in the USP14-Mut group (Figure 3(c)), demonstrating the interaction of miR-26a-5p with USP14 3′UTR. Additionally, overexpressed miR-26a-5p downregulated USP14 at the mRNA and protein levels in Müller cells under HG condition (Figures 3(d) and 3(e)). Furthermore, the influence of miR-26a-5p on the NF-*κ*B signaling pathway was assessed in HG-treated Müller cells and the results denoted that overexpressed miR-26a-5p reversed the effects of HG on the protein levels of p-I*κ*B*α*, I*κ*B*α*, NF-*κ*B (nuclear), and NF-*κ*B (cytoplasm) (Figure 3(e)). The results of immunofluorescence staining also revealed that nuclear NF-*κ*B signaling was activated after HG treatment and the alteration was reversed by miR-26a-5p mimics (Figure 3(f)). Thus, we concluded that miR-26a-5p inactivates the NF-*κ*B pathway by inhibiting I*κ*B*α* phosphorylation and NF-*κ*B nuclear translocation in HG-exposed Müller cells. In summary, miR-26a-5p targets USP14, and HG-induced activation of the NF-*κ*B pathway was suppressed by USP14 knockdown or miR-26a-5p overexpression.

### 3.4. USP14 Upregulation Reverses the Mitigative Impact of miR-26a-5p Overexpression on HG-Induced Müller Cell Dysfunction

**Figure 4:**
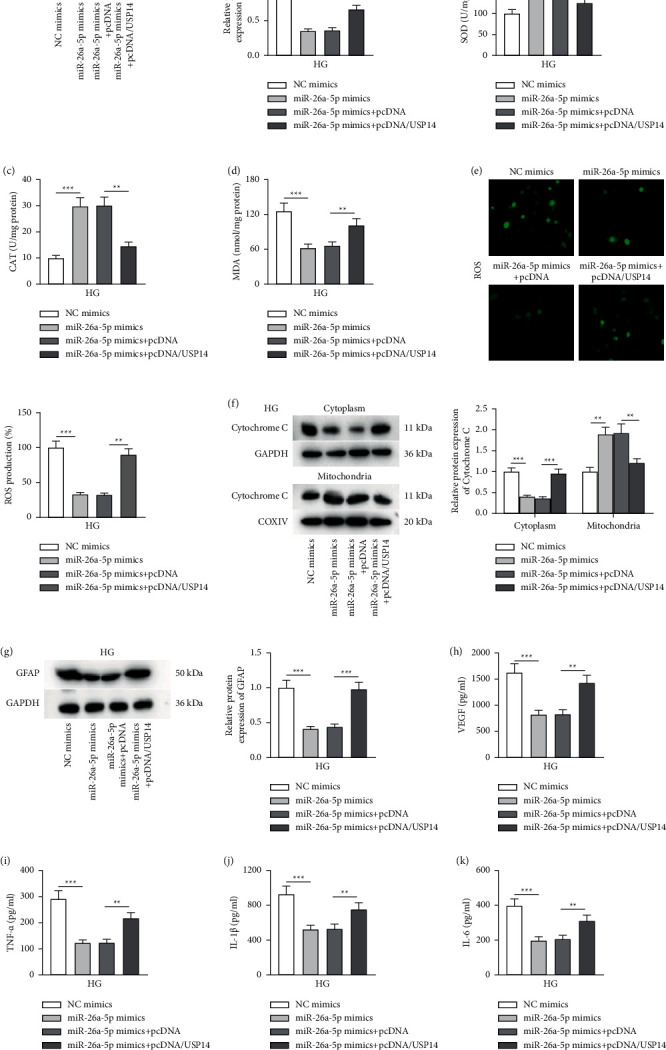
USP14 enhancement reverses the impact of miR-26a-5p upregulation on HG-induced Müller cell dysfunction. (a) Western blotting was performed to quantify USP14 protein expression in Müller cells of the NC mimics, miR-26a-5p mimics, miR-26a-5p mimics + pcDNA, and miR-26a-5p mimics + pcDNA/USP14 groups. (b–d) Measurement of SOD, CAT, and MDA levels in Müller cells of the NC mimics, miR-26a-5p mimics, miR-26a-5p mimics + pcDNA, and miR-26a-5p mimics + pcDNA/USP14 groups was performed using specific commercial kits. (e) CM-H2DCFDA staining was performed to measure ROS levels in Müller cells of the NC mimics, miR-26a-5p mimics, miR-26a-5p mimics + pcDNA, and miR-26a-5p mimics + pcDNA/USP14 groups. (f) Western blotting was performed to quantify cytochrome c protein levels in the cytoplasm and mitochondria of Müller cells of the NC mimics, miR-26a-5p mimics, miR-26a-5p mimics + pcDNA, and miR-26a-5p mimics + pcDNA/USP14 groups. (g) Western blotting for evaluating GFAP protein expression in Müller cells of the NC mimics, miR-26a-5p mimics, miR-26a-5p mimics + pcDNA, and miR-26a-5p mimics + pcDNA/USP14 groups. (h–k) ELISA was performed to measure (h) VEGF level and the accumulation of (i–k) proinflammatory cytokines (TNF-*α*, IL-1*β,* and IL-6) in Müller cells of the NC mimics, miR-26a-5p mimics, miR-26a-5p mimics + pcDNA, and miR-26a-5p mimics + pcDNA/USP14 groups. ^*∗∗*^*p* < 0.01 and ^*∗∗∗*^*p* < 0.001.

To verify whether miR-26a-5p mitigates HG-induced Müller cell injury, inflammation, and oxidative stress by targeting USP14, Müller cells were stimulated with HG followed by transfection with NC mimics, miR-26a-5p mimics, miR-26a-5p mimics + pcDNA, and miR-26a-5p mimics + pcDNA3.1/USP14, respectively. In Müller cells with HG treatment, USP14 protein level was inhibited in response to miR-26a-5p overexpression, and the change exerted by miR-26a-5p was partly reversed by USP14 overexpression (Figure 4(a)). As Figures 4(b–d) depicted, the increase in activities of antioxidant enzymes (SOD and CAT) and the decrease in the concentration of prooxidant MDA induced by overexpressed miR-26a-5p were reversed by pcDNA3.1/USP14 transfection. CM-H_2_DCFDA staining illustrated that upregulated USP14 reversed the decrease in ROS level induced by miR-26a-5p mimics in HG-exposed Müller cells (Figure 4(e)). Moreover, miR-26a-5p prevented the release of cytochrome C from mitochondria to the cytoplasm in HG-treated Müller cells, and the alteration was countervailed by overexpressed USP14 as shown by western blotting (Figure 4(f)). Furthermore, the upregulation of USP14 restored GFAP protein level and VEGF concentration that were inhibited by miR-26a-5p mimics in HG-exposed Müller cells (Figures 4(g and h)). The levels of proinflammatory cytokines (TNF‐*α*, IL‐1*β,* and IL‐6) repressed by overexpressed miR-26a-5p in HG-stimulated Müller cells were also restored after USP14 overexpression (Figures 4(i–k)). These data implied that USP14 elevation abrogates the alleviative effect of overexpressed miR-26a-5p on HG-induced oxidative stress, apoptosis, glial activation, and inflammation of Müller cells. Collectively, miR-26a-5p mitigates Müller cell dysfunction under HG treatment by targeting USP14.

### 3.5. miR-26a-5p Overexpression Alleviates Retinal Injury of Mice with DR and Alleviates Inflammation and Oxidative Stress *In Vivo*

**Figure 5:**
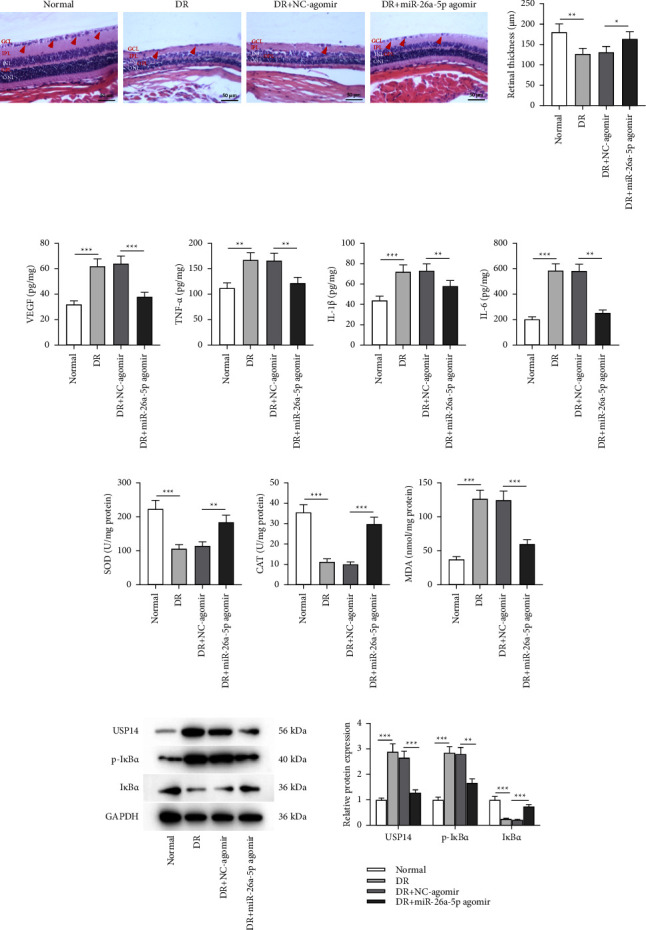
miR-26a-5p overexpression alleviates retinal injury of mice with DR by regulating the USP14/NF-*κ*B signaling. (a) H&E staining was performed to observe the pathological changes of murine retinas and to quantify retinal thickness in the normal, DR, DR + NC-agomir, or DR + miR-26a-5p agomir groups. Red arrows: the number of retinal ganglion cells. Yellow arrow: vessel-like structure; angiogenesis in the DR group. (b–e) ELISA was conducted to measure (b) VEGF level and (c–e) the accumulation of proinflammatory cytokine (TNF-*α*, IL-1*β,* and IL-6) in murine retinas of the normal, DR, DR + NC-agomir, or DR + miR-26a-5p agomir groups. (f–h) Measurement of SOD, CAT, and MDA levels in murine retinas of the normal, DR, DR + NC-agomir, or DR + miR-26a-5p agomir groups was performed using specific commercial kits. (i) Western blotting was performed to quantify the protein levels of USP14, p-I*κ*B*α,* and I*κ*B*α* in murine retinas of the normal, DR, DR + NC-agomir, or DR + miR-26a-5p agomir groups. *n* = 10/group. ^*∗*^*p* < 0.05, ^*∗∗*^*p* < 0.01, and ^*∗∗∗*^*p* < 0.001.

Whether miR-26a-5p exerts a retinal protective effect *in vivo* was validated. H&E staining of retinal tissues in indicated groups was conducted to observe histopathological changes. The control group displayed normal retinal structure and smooth retinal surfaces. In the retinas of the control group mice, each layer showed a complete and clear structure, and the cells in each layer were densely distributed and neatly arranged. However, the retinas of DR mice showed disrupted retinal structure, unclear inner and outer layers, disordered retinal cells in each layer, and angiogenesis. After injection with miR-26a-5p agomir, all these pathological changes in the retinas of DR mice were alleviated (Figure 5(a)). Quantification of retinal thickness revealed that DR induced a significant decrease in retinal thickness, and the change in DR mice was offset by miR-26a-5p overexpression (Figure 5(a)). Based on the results of ELISA, the concentrations of VEGF, TNF-*α*, IL-1*β*, and IL-6 in retinal tissues were elevated in the DR group compared to those in the normal group, and the accumulations of VEGF and proinflammatory cytokines were downregulated in the DR + miR-26a-5p agomir group compared with those in the DR + NC-agomir group (Figures 5(b–e)). The finding indicated that miR-26a-5p prevents retinal inflammation in DR mice. Consistently, the decreased activities of SOD and CAT as well as the increased MDA level were shown in the retinal tissues of DR mice when compared to those in normal mice, while the injection of miR-26a-5p agomir reversed these changes in DR mice (Figures 5(f–h)). The results demonstrated the repressive impact of miR-26a-5p on retinal oxidative stress in DR mice. Additionally, USP14 protein expression was surged in the retinal tissues of DR mice compared to that in the normal group (Figure 5(i)). Additionally, the protein level of USP14 was reduced upon the injection of miR-26a-5p agomir into DR mice (Figure 5(i)), which implied that miR-26a-5p inversely regulates USP14 *in vivo*. The regulatory role of miR-26a-5p in modulating NF-*κ*B signaling in DR mice was also verified. High p-I*κ*B*α* protein expression and low I*κ*B*α* protein expression were found in retinal tissues of DR mice compared with those in the control group, which was counteracted by miR-26a-5p agomir injection, indicating that miR-26a-5p induces the inactivation of NF-*κ*B pathway *in vivo*. To sum up, overexpressed miR-26a-5p alleviates retinal injury of DR mice and suppresses inflammation and oxidative stress *in vivo*.

### 3.6. A Schematic Diagram Revealing the Role of miR-26a-5p/USP14/NF-*κ*B Pathway in HG-Stimulated Müller Cells

**Figure 6:**
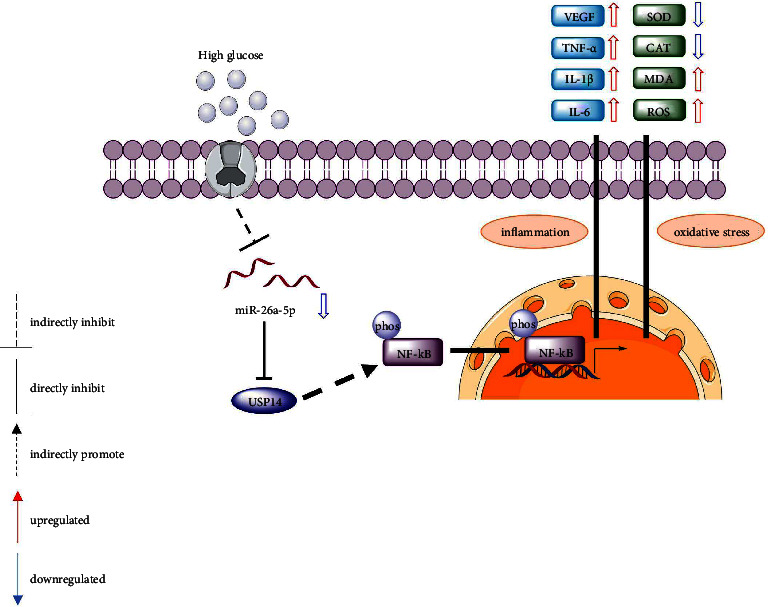
A schematic diagram revealing the role of the miR-26a-5p/USP14/NF-*κ*B pathway in HG-stimulated Müller cells.

This study revealed the protective effects of miR-26a-5p against oxidative stress and inflammation in DR *in vitro*. In Müller cells, HG exposure induces inflammation by upregulating levels of VEGF, TNF-*α*, IL-1*β*, and IL-6, and HG aggravates oxidative stress by reducing antioxidant enzymes (SOD and CAT) and increasing MDA and ROS levels. HG treatment also decreases miR-26a-5p expression in Müller cells. Moreover, miR-26a-5p directly targets USP14 and inversely regulates USP14 expression. Since USP14 can activate the NF-*κ*B signaling by promoting NF-*κ*B phosphorylation, downregulation of USP14 induced by miR-26a-5p leads to the inactivation of NF-*κ*B signaling. Activated NF-*κ*B signaling contributes to inflammation and oxidative stress in Müller cells, while the inactivation of NF-*κ*B signaling exerts the opposite effect. In conclusion, miR-26a-5p suppresses HG-triggered oxidative stress and inflammation in Müller cells by targeting USP14 and thereby inactivating the NF-*κ*B signaling (Figure 6).

## 4. Discussion

Diabetic retinopathy (DR) is a common microvascular complication of diabetes mellitus, leading to vision deficits and even irreversible blindness [1].

Müller cells are predominant glial cells in the retinal tissues, and reactive gliosis can happen in response to hyperglycemia featured by an upregulated level of GFAP (a glial activation marker) [34]. Oxidative injury and chronic inflammation are two unignorable pathogenic factors in the pathogenesis of DR [35]. During DR progression, hyperglycemic condition contributes to excessive ROS release and induces the production of inflammatory cytokines (e.g., TNF-*α*, IL-1*β*, and IL-6), which activates NF-*κ*B by promoting I*κ*B*α* and p65 phosphorylation, and p65 nuclear translocation, in turn provoking inflammatory response [36, 37]. Müller cells under HG conditions initiate subsequent oxidative stress and induce retinal inflammation by secreting various proinflammatory cytokines [27]. Thus, strategies aimed to prevent early neuroglial dysfunction and maintaining the homeostasis of Müller cells may be beneficial to treat DR or deter DR progression.

In this study, Müller cells were exposed to HG for the establishment of *in vitro* DR models. It was found that HG stimulation induced ROS accumulation, decreased antioxidative CAT and SOD concentration, and increased MDA level in Müller cells, implying the enhanced oxidative stress provoked by HG in DR *in vitro*. Cytochrome c release from the mitochondria into the cytosol is a key event in cell apoptosis [32]. HG induced cytochrome c release from the mitochondria to cytosol in Müller cells, implying that HG triggers Müller cell apoptosis. Moreover, Müller cells were activated under HG condition, as manifested by the upregulated GFAP expression. HG challenge also contributed to inflammation in Müller cells by inducing NF-*κ*B signaling activation and proinflammatory factor secretion. The abovementioned findings illustrated that HG treatment aggravates oxidative stress, inflammation, and Müller cell gliosis, which are in line with the results of previous reports [29]. Additionally, we also established the *in vivo* DR models by using streptozotocin-injected mice as previously described [38]. As a result, DR mice exhibited obvious retinal pathological injury compared to normal mice. Moreover, upregulated SOD and CAT levels, elevated proinflammatory cytokine concentration, and increased GFAP expression, as well as the activated NF-*κ*B signaling were observed in the retinal tissues of DR mice. All the data confirmed the successful establishment of *in vivo* models of DR.

Accumulating evidence has revealed that aberrantly expressed miRNAs are closely related to the initiation or development of DR since miRNAs can directly regulate genes associated with the disease [39–41]. Despite the lack of protein-coding capacity, miRNAs can modulate the expression of target mRNAs by binding to the 3′UTR of mRNAs [13]. The miRNA-mRNA regulatory mechanism is frequently found in DR. For example, miR-365 facilitates Müller gliosis, retinal injury, and oxidative stress in DR by targeting TIMP3 [42]. Downregulated miR-200b hinders Müller cell apoptosis and oxidative injury in DR via interaction with OXR1 [43]. miR-26a-5p is a miRNA that has been reported to participate in various diseases, including diabetic nephropathy [44], diabetic myocardial injury [21], diabetic cataract [45], and DR [24]. In this study, we found that miR-26a-5p was downregulated in HG-stimulated Müller cells, further confirming the potential role of miR-26a-5p in DR pathogenesis. Moreover, overexpressed miR-26a-5p reversed the impact of HG on Müller cell gliosis and cytochrome c release from the mitochondria into the cytosol. Upregulation of miR-26a-5p attenuated oxidative stress and inflammation in Müller cells under HG conditions by reducing ROS levels, enhancing SOD and CAT activities, and inhibiting the release of proinflammatory cytokines. Importantly, injection with miR-26a-5p agomir alleviated retinal histopathological alternations, inflammation, and oxidative injury in DR mice. Furthermore, the elevation of miR-26a-5p inactivated the NF-*κ*B signaling in cell models and animal models for DR. Therefore, miR-26a-5p may mitigate DR through the inactivation of the NF-*κ*B pathway.

USP14 belongs to the ubiquitin-specific processing family and can regulate various cellular activities, such as inflammation, apoptosis, oxidative stress, and proliferation [46, 47]. Moreover, emerging studies have validated its involvement in diabetes mellitus [48], DR [27], and diabetic nephropathy [49]. In this study, we found that USP14 was highly expressed in HG-treated Müller cells. Importantly, through bioinformatics prediction and experimental validation, it was confirmed that miR-26a-5p could bind to USP14 and negatively regulate USP14 expression in HG-exposed Müller cells and retinas of DR mice. Furthermore, rescue assays illustrated that USP14 upregulation reversed the mitigative effects of miR-26a-5p overexpression on oxidative stress and inflammation in Müller cells under HG conditions. The inhibitory effect of overexpressed miR-26a-5p on Müller cell gliosis and cytochrome c release from the mitochondria into the cytosol was also countervailed by upregulating USP14. Therefore, a high USP14 level aggravates DR development by enhancing HG-induced Müller cell dysfunction.

In summary, this study demonstrated that miR-26a-5p mitigates retinal injury in DR mice and represses HG-triggered Müller cell dysfunction by targeting USP14 and inactivating NF-*κ*B signaling. Our report highlights the potential application of the miR-26a-5p/USP14 axis in DR treatment. Nevertheless, to improve the accuracy of our findings, the role of miR-26a-5p in other types of DR cell models should be investigated. Additionally, the upstream molecules or other signaling pathways implicated with the miR-26a-5p/USP14 axis in DR also deserve more exploration in future studies.

## Figures and Tables

**Table 1:** Sequences for transfected plasmids.

Name of plasmids	Sequence
NC mimics	UUCUCCGAACGUGUCACGUTT

miR-26a-5p mimics	UUCAAGUAAUCCAGGAUAGGCU

sh-NC	GACCGAATATGTAAGACGCATTTCAAGAGAA
	TGCGTCTTACATATTCGGTCTTTTTT

sh-USP14	GCGACTTCAGGAAGAAATTACTTCAAGAG
	AGTAATTTCTTCCTGAAGTCGCTTTTTT

**Table 2:** Primer sequences used for RT-qPCR.

Name	Primer sequences (5′–3′)

miR-26a-5p	F: GACGGTACCTTGTCCCTGAATGTAACTCG
	R: GTTCTCGAGAAAGCAGTCCCAGCCTAAA

USP14	F: TTGCTTCGTATTCCTCGGCT
	R: TCCATAGCGGTTGCTAACTGT

U6	F: CTCGCTTCGGCAGCACA
	R: AACGCTTCACGAATTTGCGT

GAPDH	F: CAAGGTCATCCATGACAACTTTG
	R: GTCCACCACCCTGTTGCTGTAG

## Data Availability

All data analyzed or generated during the study are included either in this article or in the supplementary files.
